# Effect of MN-166 (ibudilast) on acute respiratory failure prevention in hospitalized participants with COVID-19: a randomized, double-blind, placebo-controlled phase 2 study

**DOI:** 10.1186/s12890-026-04261-8

**Published:** 2026-04-02

**Authors:** David L. Wyles, Kazuko Matsuda, Yuichi Iwaki, Emelia Klonowski

**Affiliations:** 1 Denver Health Medical Center, Denver, CO USA; 2 Medicinova, Inc , La Jolla, CA USA

**Keywords:** MN-166, COVID-19, respiratory failure, ARF, MIF, PDE4 inhibitors

## Abstract

**Background:**

MN-166 (ibudilast) is an orally available small molecule drug candidate that inhibits macrophage migration inhibitory factor and phosphodiesterase 4 and 10. The rationale for using MN-166 to prevent Acute Respiratory Failure (ARF) is based upon its cellular and molecular target actions and recent research in LPS-induced ARF animal model.

**Methods:**

In this double-blind, randomized (1:1), placebo-controlled study conducted at two centers in the United States, we evaluated MN-166 (100 mg/day: 50 mg twice daily for 7 days) compared to placebo in hospitalized participants with COVID-19 at risk for ARF. The study’s co-primary endpoints were the proportion of participants free of respiratory failure at Day 7 and the proportion of participants with at least a one-point clinical status improvement in the National Institute of Allergy and Infectious Diseases 8-point ordinal scale at Day 7. All randomized participants received a study drug and were evaluated for efficacy and safety.

**Results:**

A total of 34 participants were randomly assigned to either MN-166 or placebo (17 in each treatment group). The study achieved one of the co-primary endpoints, a statistically significant difference (*P* < 0.02) between groups related to the proportion of participants free from respiratory failure. Two-fold of participants in the MN-166 treatment group were free from respiratory failure on Day 7 versus the placebo group (70.6% vs. 35.3%; percentage difference: 35.3% [95% CI: 1.98 to 59.46]; *P* < 0.02). Sensitivity analysis showed similar differences among participants receiving interleukin-6 antibody therapy, with a statistically significant percentage difference from the placebo group (71.4% vs. 25%; 46.4%, 95% CI: 1.48 to 91.38, *P* < 0.05). MN 166 treatment was better tolerated than placebo. Two deaths were reported in the placebo group and none in the MN-166 treatment group.

**Conclusions:**

MN-166 treatment was associated with faster recovery from respiratory failure in participants with COVID-19, independent of interleukin-6 antibody therapy. MN-166 was well-tolerated with fewer adverse events compared to placebo.

**Trial registration:**

ClinicalTrials.gov NCT04429555, Registered 11 June 2020,

**Supplementary Information:**

The online version contains supplementary material available at 10.1186/s12890-026-04261-8.

## Introduction

Acute respiratory failure (ARF) is a severe manifestation of acute lung injury (ALI), characterized by acute inflammation that increases pulmonary vascular permeability and pathologically demonstrates diffuse alveolar damage. Clinically, ARF manifests hypoxemia with bilateral opacities [1]. The severe acute respiratory syndrome coronavirus-2 (SARS-CoV-2) virus’s disruption and dysregulation of the pulmonary vascular compartment was a major cause of hypoxemia in the first waves of the COVID-19 pandemic and was a major cause of respiratory failure and death [2]. Macrophage migration inhibitory factor (MIF) and phosphodiesterase (PDE)-4 inhibitors are both implicated in the pathophysiology of ARF through their roles in inflammation and immune response by different mechanisms. MIF is a pro-inflammatory cytokine that plays a crucial role in regulating the immune response. It is known to promote the release of other inflammatory cytokines and can exacerbate inflammation. In the context of ARF, elevated levels of MIF have been associated with increased severity of lung injury and inflammation. MIF can contribute to the recruitment and activation of immune cells in the lungs, leading to the disruption of the alveolar-capillary barrier and causing the accumulation of protein-rich edema fluid in the alveoli and impaired gas exchange [3–5]. PDE inhibitors have shown potential in mitigating the effects of ARF by modulating cyclic nucleotide levels within cells and maintaining the integrity of the alveolar-capillary barrier [6]. Elevated cyclic adenosine monophosphate levels reduce the production of tumor necrosis factor-α (TNF-α), interleukin 6 (IL-6), and IL-8 while promoting the production of anti-inflammatory cytokines like IL-10 [7, 8]. Meta-analyses of observational studies involving patients with COVID-19 have shown an association between using PDE4 inhibitors and improved clinical outcomes, including reduced mortality [9, 10].

MN-166, a compound that exhibits both PDE 4 and MIF inhibitory action, has shown potential in preclinical models of ALI by reducing pulmonary and systemic inflammation [11, 12]. This dual inhibition approach could be particularly beneficial in preventing ARF, where inflammation is a key pathological feature. Given the significant role of MIF and PDE4 in inflammation and its association with ARF, targeting both inhibitions represents a promising therapeutic strategy. MN-166 inhibits MIF with a half-maximal inhibitory concentration (IC_50_) of approximately 0.1 µM [13] and human PDE4A-D with IC_50_ values of 54, 65, 239, and 166 nM, respectively, and weakly inhibits other PDE families [14]. Multiple studies also have substantiated the potency and selectivity of MN-166 for specific PDE enzymes [15]. Although clinical trials of PDE4 inhibitors in ARF have yielded mixed results, subsequent analyses suggest that MN-166 may benefit patients with pulmonary inflammation in ARF [16, 17]. The rationale for the use of MN-166 for the prevention of ARF is based upon its cellular and molecular target actions and recent research in lipopolysaccharide (LPS)-induced ARF animal models [12].

This proof-of-concept pilot Phase 2 study in the US aimed to evaluate the efficacy, safety, tolerability, and biomarkers of MN-166 for prevention of severe COVID-19 in participants at risk for developing ARF.

## Methods

### Study design and participants

This study was a multicenter, randomized (1:1), double-blind, placebo-controlled, parallel-group, Phase 2 study in hospitalized COVID-19 participants at risk for developing ARF who were receiving standard care, including remdesivir and anticoagulation. This study is registered with ClinicalTrials.gov, NCT04429555, and completed. The details on the clinical study can be accessed through the ClinicalTrials.gov website at https://clinicaltrials.gov/study/NCT04429555.

**Fig. 1:**
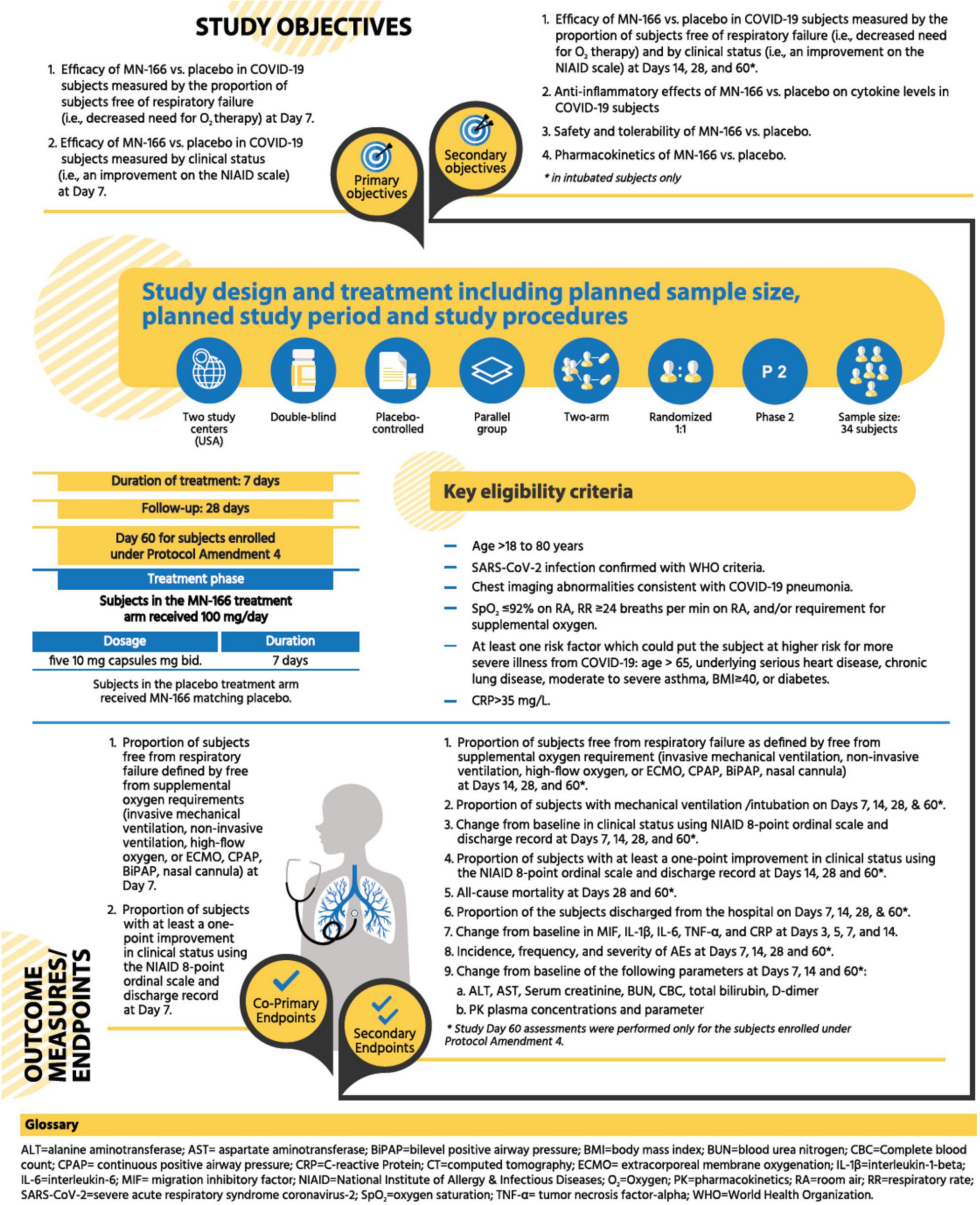
Study design, objectives and endpoints

Further details on the study design, objectives, and co-primary and secondary endpoints are presented in Fig. 1.

Eligible participants were recruited at Denver Health Medical Center (Denver, CO) and Yale-New Haven Hospital (New Haven, CT) between February 2021 and January 2022. After completion of the screening procedures, participants were enrolled in the study within 3 days and Day 1 in the study was considered ‘Baseline’ for the participants. Major inclusion criteria included hospitalized adults aged 18 to 80 years with confirmed SARS-CoV-2 infection (tested positive by polymerase chain reaction), chest imaging (radiograph, X-ray, computed tomography scan, or lung ultrasound) abnormalities consistent with COVID-19 pneumonia at risk for progression to respiratory failure based on a SpO_2_ ≤92% on room air (RA), respiratory rate ≥ 24 breaths per minute on RA, and/or requiring supplemental oxygen (O_2_), and C-reactive protein (CRP) > 35 mg/L. Participants were required to have at least one risk factor: age > 65 years, underlying serious heart disease, chronic lung disease, moderate to severe asthma, body mass index of ≥ 40, or diabetes.

Exclusion criteria included known allergies to the study drug/excipients; suspected non-COVID-19 respiratory failure; known/suspected immunosuppression with immunosuppressant medications or chemotherapeutic agents; active primary lung cancer or other malignancy metastatic to the lungs; moderate to severe liver failure (Child-Pugh score ≥ 7); lactating or pregnant at screening; or any other serious medical condition or abnormality deemed by the site primary investigator to preclude the subject’s participation in the study.

Participants were enrolled under two different versions of the study protocol. During the COVID-19 pandemic, the initial version excluded participants who were intubated or on ventilator support at the screening or baseline. However, there were few participants who were not intubated at the screening had deteriorated condition and required intubation, but continuing receiving the study drugs via nasal tube. Based on this, the principal investigator suggested to include intubated patients in the study. Additionally, there was no scientific reason to exclude the intubated patients for the study participation, therefore, we extended the inclusion criteria to include intubated patients. This exclusion criterion was removed in the second version of study protocol. However, by the time, amended study protocol was approved, after the COVID-19 outbreak was slowed down, no intubated patients were enrolled at the screening during this study. Additionally, the second version permitted the use of an IL-6 antibody therapy as a concomitant medication consistent with the evolution of the standard of care during the trial.

### Randomization and masking

The study consisted of a screening phase followed by a treatment and follow-up phase. Following the screening for eligibility, the participants were randomly assigned in a 1:1 ratio to either of the two groups: MN-166, 100 mg/day, or a matching placebo every day for 7 days. The randomization scheme was generated by an independent statistician and sent to each site’s unblinded pharmacist in a password-protected file. Randomization was stratified by the use of IL-6 antibody therapy.

Participants and all personnel involved with the conduct and interpretation of the study, including the investigators, site personnel, and sponsor staff, were blinded to the treatment codes. Randomization data were kept strictly confidential, filed securely by an appropriate group with the sponsor and accessible only to authorized persons (e.g., unblinded pharmacist) until the database is locked. To ensure that treatment allocation remains concealed to both staff and participants, the following measures were taken: (a) Active drug and placebo were identical in appearance, and (b) Drug supplies to investigational pharmacy were coded.

### Treatment administration and duration

MN-166 (five 10 mg capsules twice a day [b.i.d.]) or a matching placebo (five capsules, b.i.d.) was orally administered for 7 days. Participants who clinically progressed to require intubation and ventilator support were administered study drugs via nasogastric tube.

The rationale for the treatment duration was based on achieving steady-state plasma MN-166 concentrations within 3 days of initiating the drug. The total duration of treatment was determined to be 7 days to ensure that participants receive at least 5 days of treatment at steady-state drug levels. This approach aimed to maximize the therapeutic effect while maintaining consistent drug levels in the patient’s system, aligning with the CDC guidelines for COVID-19 management [18] and findings from Zhou et al. [19] study. Zhou et al. reported that in COVID-19 patients (*n* = 191), the median time from illness to dyspnea was 7 days, to onset of ARF was 12 days, and to ICU admission was also 12 days.

### Procedures

Upon completing the 7-day treatment phase, the participants were followed up on Day 14 and Day 28 post-baseline. On Study Day 28, the participant’s clinical status (National Institute of Allergy and Infectious Diseases [NIAID] 8-point ordinal scale (Table 1) and survival status were recorded. Some assessments were conducted on Day 60 for the participants who were enrolled under the second version of the study protocol (i.e., Protocol Amendment 4).

**Table 1:** NIAID 8-point Ordinal Scale

1. Not hospitalized, no limitations on activities.
2. Not hospitalized, limitation on activities, and/or requiring home oxygen.
3. Hospitalized, not requiring supplemental oxygen – no longer requires ongoing medical care.
4. Hospitalized, not requiring supplemental oxygen – requiring ongoing medical care (COVID-19 related or otherwise).
5. Hospitalized, requiring supplemental oxygen.
6. Hospitalized, on non-invasive ventilation or high-flow oxygen devices.
7. Hospitalized, on invasive mechanical ventilation or extracorporeal membrane oxygenation.
8. Death.

If the participant was terminated early from the study or discharged from the hospital before Day 7, the site conducted a clinical and safety assessment on that day. Those discharged before Day 7 (early) received the remainder of their study drug to take at home and were provided with a pulse oximeter to measure their O_2_ levels daily until Study Day 14. Adverse events (AEs), AE including all serious and non-serious AE, were collected from the first dose of the study drug until the end of their participation. The schedule of assessments is presented in Supplement Table S1.

### Endpoints

The co-primary endpoints of the study were: (1) the proportion of participants free from respiratory failure defined as no use of any form of supplemental O_2_, invasive mechanical ventilation, non-invasive ventilation, high-flow O_2_, extracorporeal membrane oxygenation, continuous positive airway pressure, bilevel positive airway pressure, or nasal cannula at Day 7, and (2) the proportion of participants with at least one-point improvement in clinical status on the NIAID 8-point ordinal scale and discharge record at Day 7.

### Statistical analysis

No prior data is available on which to base assumptions for sample size/power considerations. The results of this pilot study will be used to design future studies. The sample size of approximately 40 participants was deemed appropriate for this purpose. The primary analysis for the two endpoints was conducted using Pearson’s chi-square tests. Because study success was defined as success on both, each co-primary endpoint was tested using a one-sided significance level of 0.025. A supportive analysis for the two co-primary endpoints was conducted using the Mantel Haenszel test stratified by the randomization stratification variable (use of IL-6 antibody therapy). All secondary endpoints defined as proportion variables were analyzed using Pearson’s chi-square tests. Secondary endpoints defined as the change from baseline were analyzed using analysis of covariance models with the treatment group as a factor and the baseline value of the corresponding endpoint as a covariate. SAS version 9.4 or above was used for all calculations.

### Analysis population

The Full Analysis Set (FAS) comprised all randomized participants. The Safety Analysis (SA) Set comprised all randomized participants who received at least one dose of the study drug and had at least one post-dose safety assessment. The Per Protocol Analysis (PP) Set comprised the FAS participants, excluding those with major protocol violations.

Since all the randomized participants received at least one dose of study drug and none of the protocol deviations were major protocol violations, all the randomized participants were included in FAS, SA set and PP set.

## Results

### Participants disposition

**Fig. 2:**
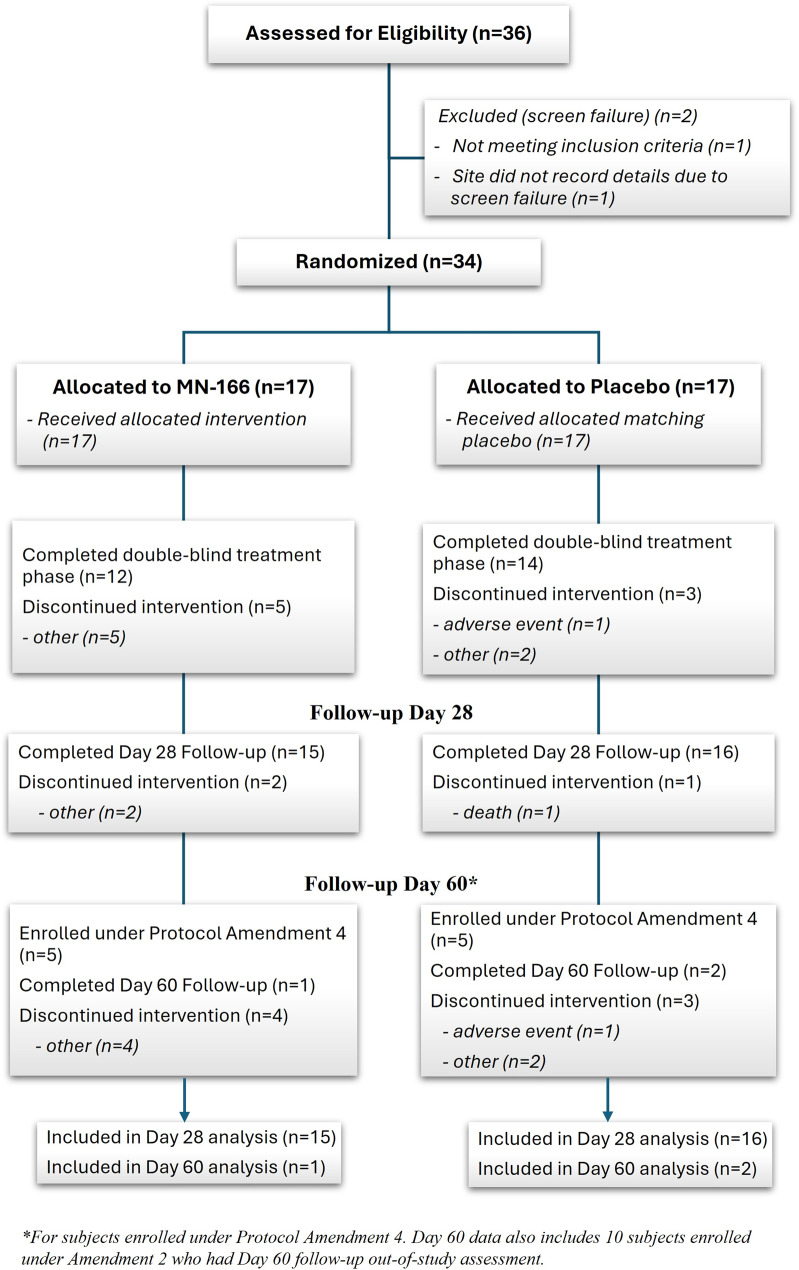
Participants’ disposition

Figure 2 presents the summary of the disposition of all enrolled participants. A total of 36 participants were screened for inclusion in the study, of whom two were classified as screen failure. One participant did not meet the inclusion criterion (no infiltrates on chest X-ray) during the Screening visit. For other screen failure participants, the site did not record the details. Thirty-four participants were enrolled and randomly assigned in a 1:1 ratio to one of two treatment groups: MN-166, 100 mg/day, or placebo (*n* = 17 each). All randomized participants were included in the FAS, PP Set, and SA Set, with 76.5% completing the 7-day treatment period and 91.2% completing the follow-up Day 28 visit. Among the 10 participants enrolled under Protocol Amendment 4, 3 (30%) participants completed the follow-up Day 60 visit. Two deaths were reported from the placebo group in the study.

### Participant baseline characteristics

Participant’s demographics are shown in Table 2. Overall, the mean age of the participants was similar across the two groups (59 ± 11 years for the MN-166 treatment group and 62 ± 12 years for the placebo group), with a balanced distribution of male and female participants across all the analysis sets. Medical history and prior and concomitant medications were recorded for all the participants.

**Table 2:** Summary of demographics and baseline characteristics

Summary Statistics	MN-166 (*N* = 17)	Placebo (*N* = 17)	Overall (*N* = 34)
Age (years)			
n	17	17	34
Mean±Standard deviation	58.71 ± 11.32	61.06 ± 12.16	59.88 ± 11.63
Median	56.00	64.00	62.50
Minimum, Maximum	40.00, 74.00	34.00, 78.00	34.00, 78.00
**Gender**,** n (%)**			
Female	9 (52.9)	8 (47.1)	17 (50.0)
Male	8 (47.1)	9 (52.9)	17 (50.0)
**Ethnicity**,** n (%)**			
Hispanic or Latino	7 (41.2)	3 (17.6)	10 (29.4)
Not Hispanic or Latino	10 (58.8)	14 (82.4)	24 (70.6)
**Race**,** n (%)**			
Black or African American	1 (5.9)	2 (11.8)	3 (8.8)
Caucasian	10 (58.8)	13 (76.5)	23 (67.6)
Native Hawaiian or Pacific Islander	1 (5.9)	0	1 (2.9)
Other	5 (29.4)	2 (11.8)	7 (20.6)
**Number of participants having one of the following risk factors ongoing at Screening**			
Cardiac disorders	2	7	9
Atrial fibrillation	1	2	3
Sinus bradycardia	0	1	1
Ventricular extrasystoles	0	1	1
Coronary artery disease	1	2	3
Arrythmia	0	1	1
Diabetes	6	7	13
Obesity (Body mass index ≥ 40)	8	4	12
Respiratory disorder**s**	8	6	14
Asthma	1	3	4
Chronic obstructive pulmonary disease	1	1	2
Cough	1	0	1
Dyspnoea exertional	1	0	1
Dyspnoea	0	1	1
Hilar lymphadenopathy	1	0	1
Pickwickian syndrome	1	0	1
Pulmonary embolism	0	1	1
Sleep apnoea syndrome	1	0	1
Tracheomalacia	1	0	1
**Number of participants required supplemental O**_**2**_ **at Baseline**,** n (%)**	17 ( 100%)	17 (100%)	34
**NIAID 8-point ordinal scale**			
Mean±Standard deviation	5.29 ± 0.59	5.41 ± 0.51	-
Median	5.00	5.00	

Abbreviations: *N* total number of participants in each treatment group, *n* number of participants in each category, *NIAID* National Institute of Allergic and Infectious Diseases, O_2_oxyygen

The proportion of the participants who required supplemental O_2_ at the baseline was the same (100% for both groups), and the mean NIAID-8 point ordinal scale at the baseline was similar (5.29 ± 0.59 for MN-166 treatment group and 5.41 ± 0.51 for placebo group). A total of 23.5% (4/17) in the MN-166 treatment group and 35.3% (6/17) in the placebo group required either non-invasive ventilation or high flow oxygen devices at the screening. None of the participants were on ECMO nor on invasive ventilators at the screening. No participants met ARF criteria at the screening.

### Efficacy

#### Co-primary outcome variables: respiratory failure and clinical status

**Fig. 3:**
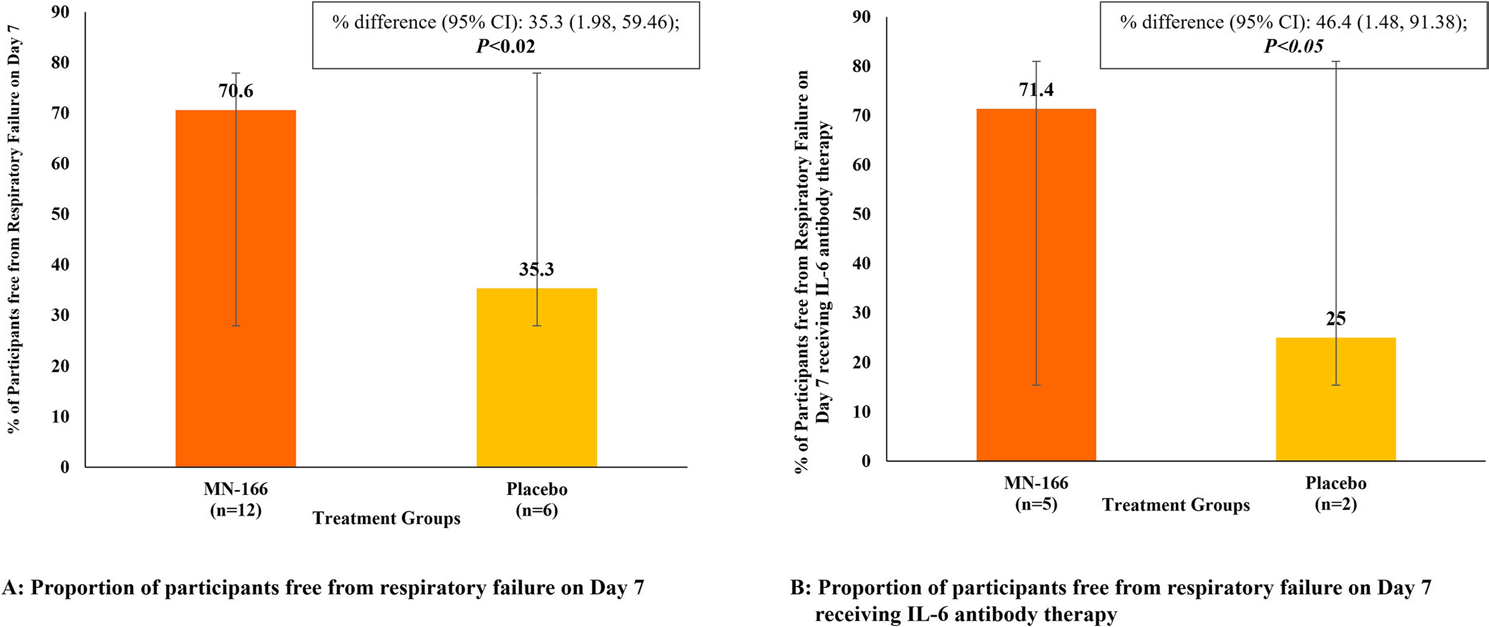
Proportion of participants free from respiratory failure on Day 7. **A**. Proportion of participants free from respiratory failure on Day 7. **B**. Proportion of participants free from respiratory failure on Day 7 receiving IL-6 antibody therapy

The study met one of its primary endpoints, with MN-166 treatment showing a statistically significant difference in the proportion of participants free from respiratory failure on Day 7 versus (vs.) the placebo group (70.6% vs. 35.5%; estimated treatment difference: 35.3%; 95% CI: 1.98 to 59.46; *P* < 0.02) (Fig. 3). These findings indicate that MN-166 treatment was beneficial and effective in faster recovery from respiratory failure in participants with COVID-19 pneumonia.

**Fig. 4:**
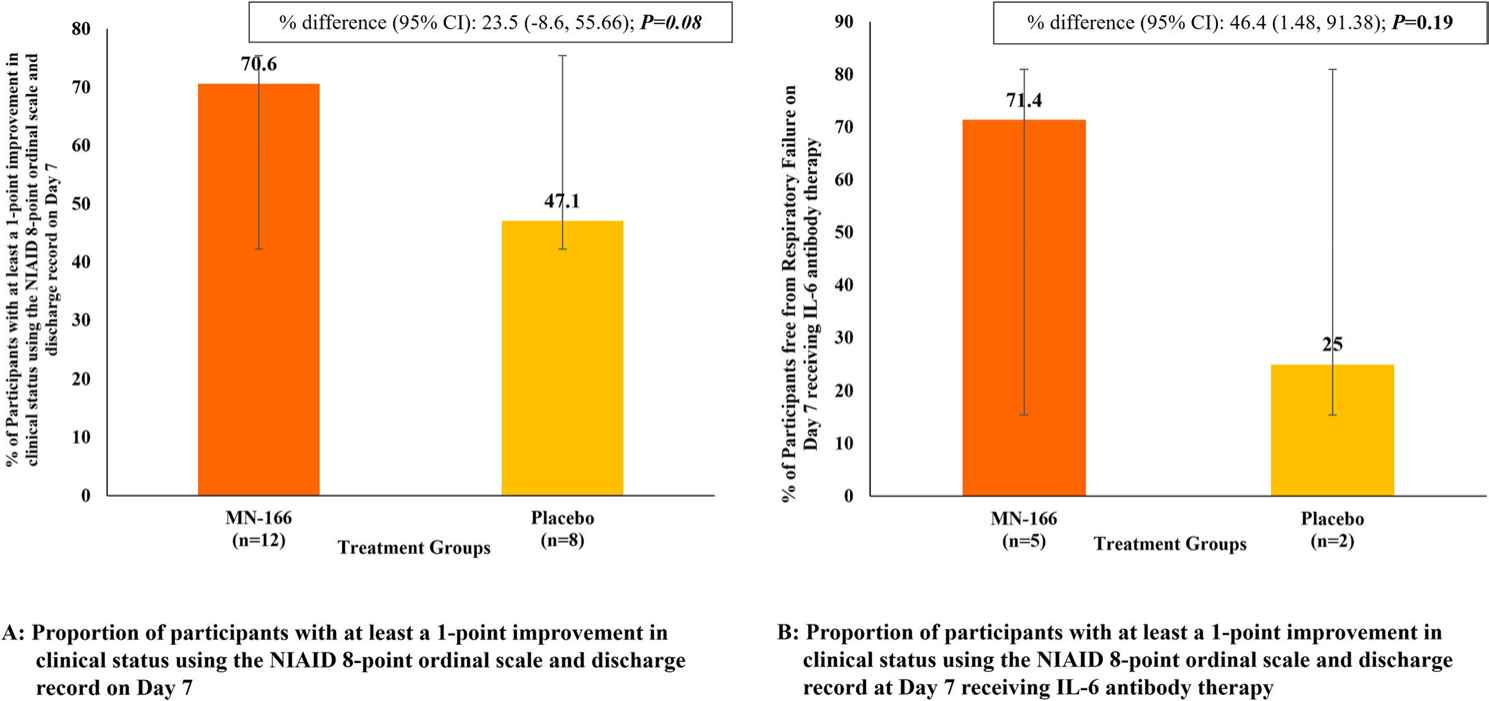
Proportion of participants with improvement in clinical status using the NIAID 8-point ordinal scale and discharge record on Day 7. **A**. Proportion of participants with at least a 1-point improvement in clinical status using the NIAID 8-point ordinal scale and discharge record on Day 7. **B**. Proportion of participants with at least a 1-point improvement in clinical status using the NIAID 8-point ordinal scale and discharge record at Day 7 receiving IL-6 antibody therapy

Numerically, a greater number of participants in the MN-166 treatment group vs. placebo demonstrated at least a 1-point improvement in clinical status using the NIAID 8-point ordinal scale at Day 7 (70.6% vs. 47.1%); however, this treatment difference (23.5%) did not achieve statistical significance (*P* = 0.08) (Fig. 4).

#### Subgroup analysis (concomitant anti-IL-6 therapy): respiratory failure and clinical status

We sought to determine if the effect of MN-166 recovered from respiratory failure or improved clinical status amongst participants receiving anti-IL-6 therapy. Amongst participants who received concomitant anti-IL-6 therapy, similar results were found favoring MN-166 treatment compared to placebo. The estimated percentage difference (46.4%; 95% CI: 1.48 to 91.38) was statistically significant (*P* < 0.05, thus indicating that participants with COVID-19 who were at risk of developing ARF responded favorably to MN-166 treatment as compared to placebo (71.4% vs. 25%) related to the proportion of participants free from respiratory failure. Results were similar for the participants who did not receive anti-IL-6 therapy (Fig. 3).

A higher percentage of participants receiving concomitant anti-IL-6 therapy during the study in the MN-166 treatment group than the placebo group showed at least 1-point improvement in their clinical status using the NIAID 8-point ordinal scale at Day 7 (71.4% vs. 25%); however, the difference was not statistically significant (*P* = 0.19). Results were similar for the participants who did not receive anti-IL-6 therapy (Fig. 4).

#### Secondary outcome variables

The results showed that there were no statistically significant (*P* > 0.05) differences observed between the treatment groups related to the proportion of participants free from respiratory failure on Day 14, Day 28, and Day 60. On Day 60, all (100%) subjects in the MN-166 treatment were free from respiratory failure, thus showing favorable results. Four participants (1 [5.9%] in the MN-166 treatment group and 3 [17.6%] in the placebo group) progressed to ARF and required supplemental oxygen by invasive ventilation or requiring ECMO on Day 7 at the end of the study drug treatment. Overall, ≤ 3 participants in either treatment group required mechanical ventilation/intubation by Days 7, 14, and 28.

It has been noted that between the treatment groups (MN-166/placebo), there was a statistically significant difference at Days 7, 14, and 28 observed in change from baseline in clinical status (NIAID-8 point ordinal scale) at Day 7 (*P*<0.0001), Day 14 (*P*<0.0001), Day 28 (*P*<0.0001), and Day 60 (*P*<0.001).

No statistically significant difference (*P*>0.05) was observed between the treatment groups related to the number of participants with at least a 1-point improvement in clinical status and discharge record at Days 14, 28, and 60. On Day 60, all (100%) subjects in both treatment groups showed improvement in their clinical status, thus showing positive outcomes.

A statistically significantly higher proportion of participants in the MN-166 treatment group were discharged by Day 7 than those in the placebo group (64.7% vs. 29.4%; *P* < 0.02), suggesting that MN-166 treatment was effective in faster recovery in participants with COVID-19 who were at risk of developing ARF.

No statistically significant difference in discharge was observed at Days 14 and 28 between placebo and MN-166. All-cause mortality was lower in the MN-166 group compared to the placebo, where 2 (11.8%) participants died from severe COVID-19 complications within 60 days in placebo group and none in MN-166 group though this difference did not reach statistical significance.

### Biomarker and CRP analysis

**Fig. 5:**
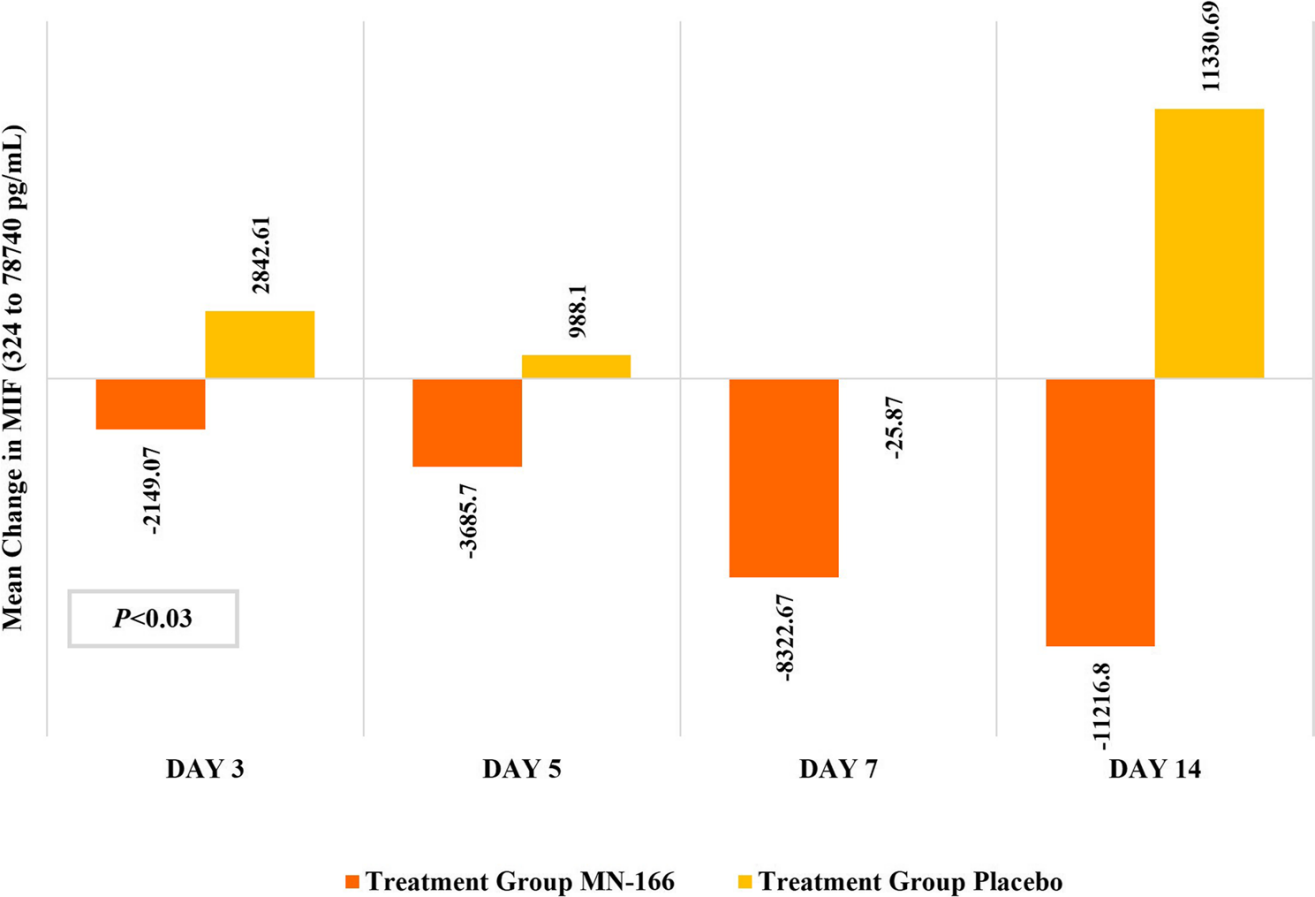
Mean change in cytokine MIF levels from baseline

The mean change in cytokine parameters (IL-1β, IL-6, monocyte chemoattractant protein-1 [MCP-1], and TNF-α) from baseline at Days 3, 5, 7, and 14 were not statistically significant. For MIF concentration, a notable reduction in the mean change in plasma MIF concentrations from baseline was observed in the MN-166 treatment group (-2149.07 [9582.80] pg/mL) at Day 3, while it was increased in the placebo group (2842.61 [5729.94] pg/mL) (Fig. 5). A positive trend was observed in the mean MIF changes from baseline within the MN-166 treatment group, with P values ranging from 0.03 to 0.09.

**Fig. 6:**
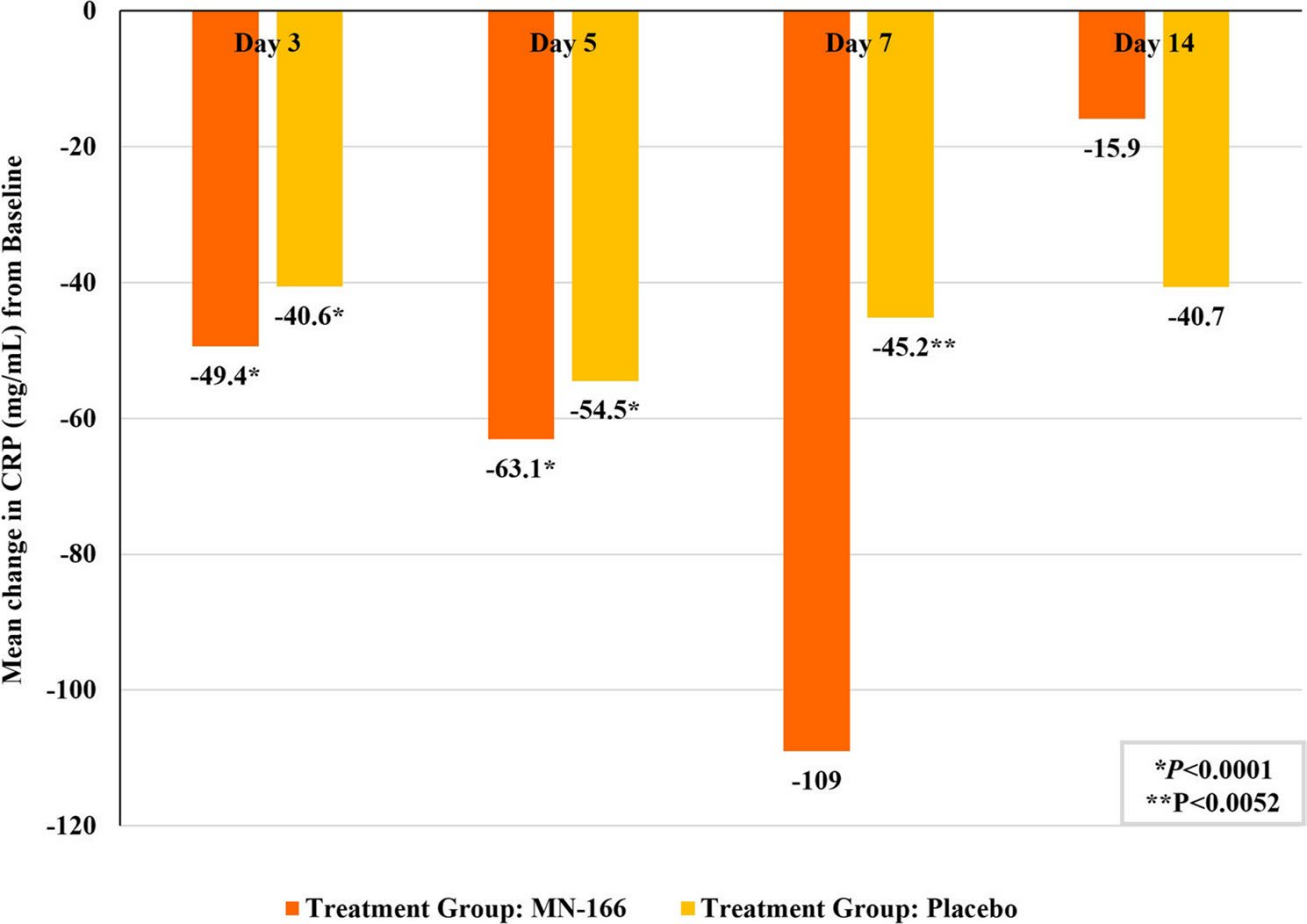
Mean change in C-reactive protein from baseline

There were statistically significant reductions in the mean CRP values from Baseline to Day 3 (*P* < 0.0001), Day 5 (*P* < 0.0001), and Day 7 (*P* < 0.0052 in the placebo group) within the treatment groups (Fig. 6). Serum CRP Day 14 samples were only obtained from limited participants who were still in the hospital (*n* = 5 from MN-166 and *n* = 7 from placebo), and the mean CRP levels on Day 14 were higher than on Days 3, 5, and 7 in both groups.

### Safety

The findings of the study indicate that the MN-166 treatment was well-tolerated and had a better safety profile compared to the placebo.

Overall, 27 (79.4%) of 34 evaluable participants reported a total of 99 AEs − 13 (76.5%) of 17 participants in the MN-166 treatment group reported a total of 37 events; and 14 (82.4%) of 17 participants in the placebo group reported a total of 62 events. Of the 99 AEs reported, 93 were treatment-emergent adverse events (TEAEs), 19 were severe TEAEs, and 12 TEAEs led to the death of 2 participants in the placebo group. A greater number of TEAEs occurred in the placebo group than in the MN-166 treatment group (56 vs. 37). Among these, 12 events were considered study drug related. All the drug related AEs were mild to moderate in severity.

More SAEs were reported in the placebo group than in the MN-166 treatment group.

Overall, 8 (8/34 = 23.5%) participants reported 19 SAEs, with 13 SAEs reported in the placebo group and 6 SAEs reported in the MN-166 treatment group. Of 9 life-threatening SAEs, 8 SAEs were reported from 3 (17.6%) participants in the placebo group, and 1 SAE was reported from 1 (5.9%) participant in the MN-166 treatment group.

There was no death s reported in the MN-166 treatment group, while 2 (11.8%) participants in the placebo group died due to complications of severe COVID-19 within 60 days of receiving the study drug (Table 3).

**Table 3:** Summary of Adverse Events by Treatment

Any adverse events	13 (76.5) 37	14 (82.4) 62	27 (79.4) 99
Any serious adverse events	3 (17.6) 6	5 (29.4) 13	8 (23.5) 19
Any treatment-emergent adverse events	13 (76.5) 37	14 (82.4) 56	27 (79.4) 93
Drug-related treatment-emergent adverse events	7 (41.2) 7	4 (23.5) 5	11 (32.4) 12
Severe treatment-emergent adverse events	3 (17.6) 7	4 (23.5) 12	7 (20.6) 19
Treatment-emergent adverse events Leading to Early Termination	0	1 (5.9) 1	1 (2.9) 1
Treatment-emergent adverse events Leading to Death	0	2 (11.8) 12	2 (5.9) 12
**Incidence of TEAEs Occurring in ≥ 10% of Participants**			
**Preferred Term**	**MN-166** (N = 17) **n (%) E**	**Placebo** **(N = 17)** **n (%) E**	**Overall** **(N = 34)** **n (%) E**
Leukocytosis	0	3 (17.6) 3	3 (8.8) 3
Bradycardia	7 (41.2) 7	3 (17.6) 3	10 (29.4) 10
Sinus bradycardia	2 (11.8) 2	1 (5.9) 1	3 (8.8) 3
Hyperglycaemia	0	3 (17.6) 3	3 (8.8) 3
Constipation	0	2 (11.8) 2	2 (5.9) 2
Diarrhoea	3 (17.6) 3	2 (11.8) 3	5 (14.7) 6
Transaminases increased	1 (5.9) 1	4 (23.5) 4	5 (14.7) 5
Headache	1 (5.9) 1	2 (11.8) 2	3 (8.8) 3
Acute kidney injury	0	3 (17.6) 3	3 (8.8) 3
Hypoxia	0	2 (11.8) 2	2 (5.9) 2
Pneumonia	2 (11.8) 3	2 (11.8) 3	4 (11.8) 6
Respiratory failure	2 (11.8) 2	3 (17.6) 3	5 (14.7) 5
Hypotension	0	2 (11.8) 2	2 (5.9) 2

Abbreviations: *E* number of events reported within each treatment group, *n *number of participants who experienced the specific event; *TEAE* treatment-emergent adverse event.

Body system totals were not necessarily the sum of the individual AEs since a patient could have reported more than one AE in the same body system. AEs were coded into System Organ Class and Preferred Term using MedDRA version 25.0.

Percentages were computed using N provided in the column header

At all study visits, no notable trend or marked differences observed in either treatment group in mean changes over time or shifts from the baseline in clinical laboratory parameters, vital signs and electrocardiogram. Abnormal laboratory values were recorded as AEs.

## Discussion

The study findings demonstrate that MN-166 may be effective in faster recovery from respiratory failure and prevent clinical worsening amongst participants with COVID-19 at risk for developing ARF as additionally a significantly higher proportion of participants in the MN-166 group were able to be discharged by Day 7 than those in the placebo group.

The current Phase 2 study depicted the superior efficacy of MN-166 in severe COVID-19 participants with a lower incidence of AEs than the placebo group. MN-166 was well-tolerated and demonstrated favorable safety with fewer AEs reported compared to the placebo group. This lower incidence of AEs in MN-166 may be attributed to the clinical improvements observed in participants receiving MN-166. Importantly, MN-166 treatment did not result in an increase in side effect symptoms relative to the placebo.

MN-166 was expected to prevent ARF in COVID-19 patients by inhibiting PDE-4 and MIF-mediated cytokine toxicity in severe cases [12–15, 20]. In an experimental mouse model of LPS-induced neonatal ARF, ibudilast attenuated the secretion of inflammatory cytokines, TNF-α, IL-1β, IL-6, and MCP-1 by inactivating the cytokine stimulation axis while also significantly reducing LPS-induced cell apoptosis in lung tissue [12, 21]. The current study observed a positive trend with the reduction with mean serum MIF level from baseline at Day 3. Reduction of serum MIF level by MN-166 treatment was not expected as an allosteric MIF inhibitor; however, it is consistent with the previous study conducted with MN-166 [12]. Although it was not expected by the mechanism of action, this observation is crucial as elevated levels of serum MIF could lead to severe inflammation and poor outcomes in patients with ARF [4, 22]. It also indicates there is target engagement between MN-166 and MIF suggesting that the mechanism of action of MN-166 is a reduction in MIF as well as inhibition of PDE.

Significant reductions in the mean CRP values from Baseline were observed in both groups at Days 3 and 5, which reflect clinical improvement. In both groups, the mean CRP levels on Day 14 were higher than on Days 3, 5, and 7. It was most likely because the Day 14 samples were collected only from limited subjects who remained in the hospital due to insufficient recovery and were not collected from the participants who were discharged after clinical improvement with a reduction of CRP level.

Anti-inflammatory therapies have played a crucial role in managing ARF in COVID-19 patients. Previous studies have shown that dexamethasone [23], tocilizumab (IL-6 antibody) [24], and baricitinib (a JAK inhibitor) [24] can reduce the need for mechanical ventilation, enhance recovery times, and improve survival rates in critically ill patients. The study by Palacios-Moguel P et al. [25] focused on the unique phenotype of ARDS in COVID-19 patients compared to those with non-COVID-19 ARDS patients [25]. These patients were typically younger males with higher BMI and fewer comorbidities. COVID-19 ARF was more severe, with higher admission and mortality rates, frequent organ dysfunction, and increased need for renal replacement therapy. Similar to MN-166, roflumilast has demonstrated immune balancing effects by effectively decreasing lung edema and improving respiratory parameters in experimental models [5]. The pathophysiologic mechanisms described by Palacios-Moguel et al. [25] that derive COVID-19 ARDS are likely active in other forms of lung injury, so it is possible that MN-166 might be effective in other severe acute inflammatory lung conditions, and its treatment effect was observed in chlorine gas-induced-lung injury ovine model study [11].

Antiviral agent, nirmatrelvir/ritonavir (Paxlovid), is available for treating mild-to-moderate COVID-19 in patients who are at increased risk of progression to severe COVID-19 and must be taken within 5 days of the occurrence of the symptoms [26]. Unlike the antiviral agents, MN-166 is not SARS-CoV2-targeted but rather treatment for the symptoms by modulating upstream inflammatory pathways through MIF and PDE4 inhibition. Having both selective PDE4 inhibits effects and the ability to reduce inflammatory MIF levels, MN-166 stands out as a promising intervention fulfilling the requirements for treating inflammation-based pathologies and preventing hyperinflammation in severe COVID-19 participants at risk of developing COVID-19 pneumonia or ARF. This mechanism indicates the potential utility of MN-166 in COVID-19 and in severe inflammatory lung injury from other causes, including other viruses, bacteria or chemical exposure [27]. With supportive phase 2 data on established safety profile of MN-166, availability of oral formulation, and immunomodulatory rather than antiviral action, MN-166 can be used in outpatient settings to prevent acute respiratory failure. Preclinical evidence with other PDE4 inhibitors also supports early-phase outpatient investigation.

### Study limitations and future directions

This study did not meet both co-primary endpoints, which may have been due to the small sample size. Comparable studies demonstrating the effect of anti-inflammatory therapies such as steroids and tocilizumab for COVID-19 were much larger in size, thus limiting generalizability. The study was conducted during a period when specific COVID-19 variants were circulating, which may differ from current variants. This could affect the generalizability of the results in the present situation.

Since the time of the study, population immunity has increased due to widespread vaccination and natural infection. This change in immunity levels could influence the effectiveness of MN-166. Considering the complex and multifactorial nature of ARF and given the promising results of this study, testing the efficacy of MN-166 in a larger cohort is warranted.

This was the first study targeting severe COVID-19 pneumonia patients receiving multiple concomitant medications, therefore, we limited the treatment duration to 7 days to avoid any unforeseen drug-drug interactions between MN-166 and other medications. Observing that MN-166 treatment showed same better outcomes in participants using IL-6 antibody therapy (with no drug-drug interaction concerns), along with accumulated safety data (up to 100 mg/day for up to 2 years of treatment), consideration may be given to extending the treatment duration in future studies in patients at risk of developing ARF from pneumonia or related respiratory conditions. The availability of other effective therapies, such as dexamethasone and remdesivir, has increased. Future studies should consider evaluating MN-166 in combination with these therapies to determine potential synergistic effects.

## Conclusions

This phase 2 study showed that MN-166 effectively promotes faster recovery from respiratory failure and facilitates earlier discharge in participants with severe COVID-19, with fewer AEs, SAEs and deaths compared to the placebo group. MN-166 was safe and well-tolerated, even in those receiving multiple other treatments. Its potent anti-inflammatory effects and modulation of key inflammatory pathways make it a promising candidate for faster recovery from severe COVID-19 and prevent ARF. Further randomized studies are needed to confirm these findings and explore the long-term prognosis of MN-166 in severe COVID-19 and ALI from other pathophysiology.

## Supplementary Information

Supplementary Material 1.

## Data Availability

The datasets used or analyzed during this study are not publicly available to maintain confidentiality but could be available from the corresponding author on reasonable request.

## Abbreviations

AE: Adverse Events
ALI: Acute Lung Injury
ARF: Acute Respiratory Failure
BMI: Body Mass Index
CRP: C-reactive Protein
ECG: Electrocardiogram
ECMO: Extracorporeal Membrane Oxygenation
FAS: Full Analysis Set
LPS: Lipopolysaccharide
MIF: Macrophage Migration Inhibitory Factor
NIAID: National Institute of Allergy and Infectious Diseases
O_2_: Oxygen
PDE: Phosphodiesterase
PP: Per Protocol
RA: Room Air
SA: Safety Analysis
SAE: Serious Adverse Events
TEAE: Treatment-emergent Adverse Events
