# Supplementary material for: Effect of MN-166 (ibudilast) on acute respiratory failure prevention in hospitalized participants with COVID-19: a randomized, double-blind, placebo-controlled phase 2 study
Source: BMC Pulm Med. 2026 Apr 2;26:218. doi: 10.1186/s12890-026-04261-8 (PMC13170009; doi:10.1186/s12890-026-04261-8)
Supplement: Supplementary file 1 — Supplementary Material 1. [file 12890_2026_4261_MOESM1_ESM.docx]

**Supplementary Data**

Supplement Table S1: Schedule of Assessments

| Phase | Screening | Double-Blind Treatment Phase^a,b^ | | | | | | | Follow-up | | |
| --- | --- | --- | --- | --- | --- | --- | --- | --- | --- | --- | --- |
| Study Day | Up to Day -3 | Day 1 BL | Day 2 | Day 3 | Day 4 | Day 5 | Day 6 | Day 7 | Day 14^a^ (± 3) | Day 28 (±3) | Day 60^k^ (±7) |
| Informed consent | X |  |  |  |  |  |  |  |  |  |  |
| Inclusion/exclusion criteria review | X |  |  |  |  |  |  |  |  |  |  |
| Brief physical exam | X | X |  | X |  | X |  | X | X |  |  |
| Clinical status using the NIAID scale | X | X | X | X | X | X | X | X | X | X | X |
| Vital signs (BP, RR, HR, Temp) | X | X | X | X | X | X | X | X | X |  | X^c^ |
| O_2_ therapy status and SpO_2_ | X | X | X | X | X | X | X | X | X | X | X |
| 12-lead ECG | X | X^d^ |  | X^d^ |  | X^d^ |  | X^d^ |  |  | X^c^ |
| Biomarker plasma samples: MIF, IL-1β, IL-6, TNF-α | X^e^ | X |  | X |  | X |  | X | X |  |  |
| PK blood sample^f^ |  | X |  | X |  | X |  |  |  |  |  |
| CBC, CMP, CRP, D-dimer, PT, INR | X^g^ | X |  | X |  | X |  | X | X |  | Xi |
| Randomization^h^ | X |  |  |  |  |  |  |  |  |  |  |
| Administer study drug |  | X^i^ | X | X | X | X | X | X |  |  |  |
| Adverse event review |  | X | X | X | X | X | X | X | X | X | X |
| Prior/Concomitant medication review | X | X | X | X | X | X | X | X | X | X | X |
| Record survival status^j^ |  |  |  |  |  |  |  |  | X | X | X |

Abbreviations: BL=baseline; BP=blood pressure; CBC=complete blood count; CMP=comprehensive metabolic panel; CRP=C‑reactive protein; ECG=electrocardiogram; HR=heart rate; IL-1β=interleukin-1 beta; IL-6=interleukin-6; INR=international normalized ratio; MIF=macrophage migration inhibitory factor; NIAID=National Institute of Allergy and Infectious Diseases; O_2_=oxygen; PK=pharmacokinetic; PT=prothrombin time; RR=respiratory rate; SpO_2_=peripheral capillary oxygen saturation; Temp=temperature; TNF‑α=tumor necrosis factor-alpha.

Notes.

1. Telephone follow-up if the participant was not in the hospital. If the participant was no longer hospitalized or could not return due to COVID-19 restrictions, a telephone follow-up was conducted to record O_2_ usage/levels and a review of adverse events and concomitant medications. All other assessments were optional.
2. If the participant prematurely discontinued the study or was discharged from the hospital on Days 2, 4, or 6, collect biomarker samples in addition to other scheduled assessments. If the participant prematurely discontinued the study or was discharged on Days 1, 3, 5, or 7, conduct the study assessments scheduled on those days.
3. Vital signs, ECG, and safety labs were done in intubated participants only.
4. 12-lead ECG was done between 2-4 hours after AM dose.
5. Biomarker blood sample collection at the screening was optional if the Screening and Day 1 assessment occurred on the same day.
6. Collected PK samples on Day 1 in the AM before study drug dosing and at 2, 4, 8, and 12 hours (predose of PM dose); Day 3 and Day 5 before AM study drug dosing. PK samples were collected for 8 participants only.
7. Serum beta-human chorionic gonadotrophin (β-hCG) for premenopausal women.
8. Participants were randomized during the Screening Phase after all the screening assessments were completed and the participant was considered eligible for the study or on Day 1.
9. The first dose of the study drug started either at AM or PM.
10. Survival status was captured on the NIAID questionnaire.
11. Day 60 follow-up assessment is only for participants enrolled under Amendment 4.
